# Targeting Signaling Pathways in Inflammatory Breast Cancer

**DOI:** 10.3390/cancers12092479

**Published:** 2020-09-01

**Authors:** Xiaoping Wang, Takashi Semba, Lan Thi Hanh Phi, Sudpreeda Chainitikun, Toshiaki Iwase, Bora Lim, Naoto T. Ueno

**Affiliations:** 1 Section of Translational Breast Cancer Research, Department of Breast Medical Oncology, The University of Texas MD Anderson Cancer Center, Houston, TX 77030, USA; TSemba1@mdanderson.org (T.S.); LTPhi@mdanderson.org (L.T.H.P.); SChainitikun@mdanderson.org (S.C.); TIwase@mdanderson.org (T.I.); BLim@mdanderson.org (B.L.); 2 Morgan Welch Inflammatory Breast Cancer Research Program and Clinic, The University of Texas MD Anderson Cancer Center, Houston, TX 77030, USA; 3 The University of Texas MD Anderson Cancer Center UT Health Graduate School of Biomedical Sciences, Houston, TX 77030, USA

**Keywords:** inflammatory breast cancer, signaling pathways, tumor microenvironment, targeted therapy, clinical trials

## Abstract

**Simple Summary:**

Inflammatory breast cancer (IBC) is the most lethal and aggressive form of breast cancer; it is highly likely to spread to other sites in the body. There is an urgent need to establish novel treatment strategies to reduce IBC recurrence and metastasis. The aim of this work is to provide a comprehensive overview of signaling pathways in IBC, covering understanding of their function in IBC tumor cells and cells surrounding tumor, and clinical efforts to target these pathways for patients with IBC. The findings described in this work will help guide the development of effective therapies through preclinical and clinical research, eventually improving the treatment of patients with IBC.

**Abstract:**

Inflammatory breast cancer (IBC), although rare, is the most aggressive type of breast cancer. Only 2–4% of breast cancer cases are classified as IBC, but—owing to its high rate of metastasis and poor prognosis—8% to 10% of breast cancer-related mortality occur in patients with IBC. Currently, IBC-specific targeted therapies are not available, and there is a critical need for novel therapies derived via understanding novel targets. In this review, we summarize the biological functions of critical signaling pathways in the progression of IBC and the preclinical and clinical studies of targeting these pathways in IBC. We also discuss studies of crosstalk between several signaling pathways and the IBC tumor microenvironment.

## 1. Introduction

Inflammatory breast cancer (IBC) is the most lethal type of breast cancer, with a high rate of metastasis [1,2,3,4]. Only 2–4% of breast cancer cases in the United States are classified as IBC, but IBC results in a higher rate of death compared with non-inflammatory breast cancer (non-IBC). The median survival of patients with IBC is short (2.9 years) compared with locally advanced breast cancer (LABC, T4; 6.4 years) and non-T4 breast cancer (longer than 10 years) [5]. The 5-year overall survival rates of patients with IBC is less than 55% [6,7,8]. The diagnosis of IBC is based on the clinical characteristics, including erythema, edema, and/or orange peel of the breast; rapid onset of symptoms, and T4d stage, according to the American Joint Committee on Cancer staging system [9]. Although many efforts have been made, molecular or pathological diagnostic criteria for IBC have not yet been identified. The molecular subtypes of IBC patients include hormone receptor-positive (HR+, positive for estrogen and/or progesterone receptors)/HER2+ (14.8%), HR+/HER2-negative (HER2−; 35.7%), HR−/HER2+ (23.1%), and triple-negative (TN-IBC, negative for estrogen and progesterone receptors and HER2; 26.4%) [7]. Among these IBC subtypes, TN-IBC carries the worst prognosis.

**Figure 1:**
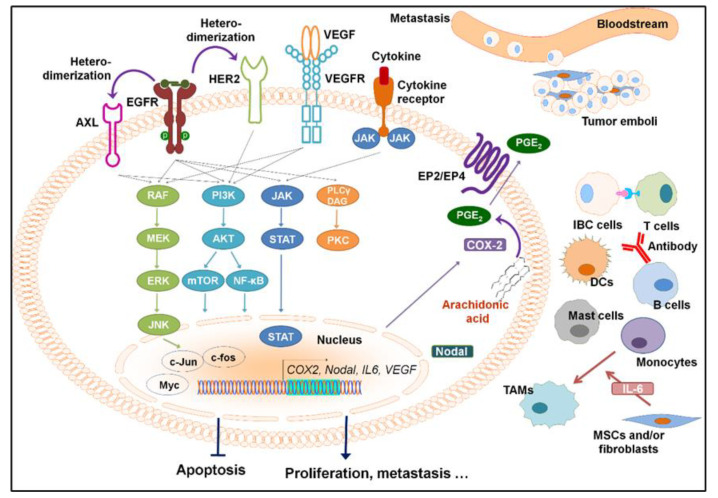
Overexpressed or activated signaling pathways in inflammatory breast cancer and the tumor microenvironment. The presence of tumor emboli, in which cells pack together to form a tumor cell cluster, is a hallmark of IBC cells. DCs, dendritic cells; IBC, inflammatory breast cancer; MSCs, mesenchymal stem cells; TAMs, tumor-associated macrophages.

The standard treatment for IBC is trimodality therapy, which consists of chemotherapy, surgery, and radiation. Although this therapeutic approach has significantly improved patient survival, the median survival of IBC patients remains poor [10]. We are in need of novel targeted therapies derived via understanding the molecular mechanism of IBC and identifying novel targets to improve patients’ outcomes. Over the past two decades, researchers have identified some molecular changes that play important roles in IBC. These include loss of WNT1-inducible-signaling pathway protein 3 (WISP3) [11]; overexpression of epidermal growth factor receptor (EGFR) [12], HER2 [13], TIG1/Axl [14], E-cadherin [15], *Ras* homolog gene family member *C* (*RhoC*), and GTPase [16]; overexpression of inflammatory mediators Janus kinase (JAK)/signal transducers and activators of transcription (STAT) [17,18], nuclear factor kappa B (NF-κB) [19,20,21], and Cyclooxygenase-2 (COX-2) [20,22] as well as angiogenic factors [23,24] and translation initiation factor eIF4GI [25]; and enrichment of the cancer stem cell (CSC) population [26]. Recently, the tumor microenvironment (TME), which includes T cells, tumor-associated macrophages (TAMs), fibroblasts, mast cells, and mesenchymal stem cells (MSCs), has been shown to play critical roles in promoting IBC aggressiveness. TAMs enhance IBC cell migration [27]. The co-injection of MSCs with IBC cells can drive IBC clinical phenotypes, including increased skin invasion and metastasis [28]. The TME also contributes to the response of IBC patients to neoadjuvant chemotherapy (NAC). It has been shown that the presence of tumor-infiltrating lymphocytes was increased in the tumors of IBC patients who had a pathological complete response (pCR) to NAC. In contrast, mast cell infiltration was associated with poor response to NAC in patients with IBC [29]. Here, we summarize the biological functions of several critical signaling pathways in the progression of IBC, their crosstalk with the IBC TME, and the up-to-date preclinical and clinical studies of targeting these pathways in IBC (Figure 1).

## 2. EGFR

### 2.1. Biological Functions of EGFR Pathway in IBC

EGFR, also called ErbB1 or HER1, belongs to ErbB family of receptors, which also includes HER2, HER3, and HER4. Upon binding to its ligands, EGFR forms an active homodimer or heterodimerizes with other ErbB family members such as HER2, activating downstream pathways involved in cell growth, proliferation, migration, and differentiation such as the mitogen-activated protein kinase (MAPK), AKT, c-Jun N-terminal kinase (JNK), and phosphoinositide phospholipase C/protein kinase C (PLC/PKC) signaling pathways. EGFR is overexpressed in IBC and other types of breast cancer [12,30]. EGFR expression independently predicts a high recurrence rate and shorter survival duration in patients with IBC. The 5-year overall survival rate of IBC patients with EGFR-positive disease is significantly lower than that of patients with EGFR-negative disease [12].

EGFR signaling regulates tumor growth through its downstream AKT and ERK pathways. EGF and AREG ligands can activate EGFR signaling and promote the proliferation of IBC SUM149 cells in vitro [31]. In contrast, inactivation of the EGFR pathway using tyrosine kinase inhibitors gefitinib and erlotinib or EGFR knockdown suppressed the proliferation of IBC cells through the MAPK/ERK pathway as well as tumor growth in vivo [32,33]. Patients with IBC have a high tendency of distant metastasis. Erlotinib treatment reduces the invasion of IBC cells, reduces the expression of epithelial-to-mesenchymal transition (EMT) markers, and inhibits spontaneous lung metastasis in vivo [33].

Patients with IBC have inflammatory clinical characteristics such as diffuse erythema and edema of the breast, and COX-2 is an important inflammatory molecule in IBC. Wang et al. [22] demonstrated that the expression of EGFR and COX-2 correlate with each other in IBC tumor biopsy samples and that EGFR regulates COX-2 expression in IBC cells.

IBC patients have high expression of aldehyde dehydrogenase 1 (ALDH1), a CSC marker, which correlates with metastasis and worse patient outcome [26]. It has been reported that the EGFR pathway regulates CSC in IBC, as indicated by the reduced formation of primary and secondary mammospheres, and reduces CD44+/CD24− and ALDH+ populations—a hallmark of breast CSC—in IBC cells by the depletion of EGFR or inhibition of EGFR signaling [22]. The regulation of CSC by EGFR is mediated by COX-2 and nodal signaling in IBC.

The EGFR pathway regulates the crosstalk between tumor cells and TME. Lacerda et al. [27] showed that the co-injection of MSCs with IBC SUM149 cells significantly increased skin invasion and metastasis in vivo, which are the clinical features of IBC. They also found a higher expression of phosphorylated EGFR (pEGFR) and more metastasis in tumors produced by the co-injection of SUM149 with mesenchymal stem cells compared with tumors grown from SUM149 cells only; the EGFR inhibitor erlotinib abrogated these effects [28]. In addition, the researchers showed that pEGFR expression in the stroma correlates with its expression in tumor cells in IBC patients but not in non-IBC patients [28]. TAMs are another main member of the TME and contribute to tumor progression and invasion by inducing immunosuppression, mediating tumor matrix remodeling, and supporting vascular potency [34,35,36]. Invasive tumor cells can migrate together with macrophages in primary mammary tumors in response to EGF and colony-stimulating factor 1 (CSF-1), which can be blocked by inhibiting either EGFR or CSF-1 signaling [37]. Macrophages also help tumor cells enter blood vessels; however, the inactivation of EGFR signaling blocks this process [38]. Treatment of human THP1 monocytes with erlotinib inhibited the polarization of M2 macrophages from monocytes (N.T. Ueno, unpublished data).

### 2.2. Targeting EGFR Pathway in IBC

Clinical trials of EGFR-targeted therapy for breast cancer patients have not shown clear clinical benefits. These clinical trials were summarized in our previous review article [39]. Here, we will focus on preclinical and clinical studies of targeting EGFR with erlotinib, panitumumab, and neratinib in IBC.

Erlotinib binds to the intracellular domains of EGFR and inhibits kinase activity and downstream signaling. In vitro studies indicated that erlotinib treatment inhibited IBC cell proliferation, anchorage-independent growth, cell motility, the COX-2 inflammatory pathway, and CSC marker-bearing cells in IBC [22,33]. In vivo, erlotinib reduced the growth of IBC primary tumors and metastasis to the lung [33].

Panitumumab is a humanized anti-EGFR monoclonal antibody. It binds to EGFR and blocks the binding of EGF ligand, thus inactivating EGFR signaling. There have been two clinical studies of panitumumab in combination with neoadjuvant chemotherapy in patients with IBC (ClinicalTrials.gov Identifier: NCT01036087 and NCT02876107; Table 1). NCT01036087 (Phase II Study of Panitumumab, Nab-paclitaxel, and Carboplatin for Patients With Primary Inflammatory Breast Cancer (IBC) Without HER2 Overexpression) is a single-arm phase 2 study of neoadjuvant therapy with panitumumab, nab-paclitaxel, and carboplatin (PNC) followed by 5-fluorouracil, epirubicin, and cyclophosphamide (FEC) in patients with newly diagnosed, HER2− primary IBC [40]. Patients received one dose of panitumumab followed by four cycles of PNC weekly and then four cycles of FEC every 3 weeks. The pCR rate was 28% in all evaluable patients, 42% in TN-IBC patients, and 14% in HR+/HER2− IBC patients. The treatment regimen had acceptable hematological and dermatological toxic effects, and there were no treatment-related deaths. A correlative study identified that pEGFR and COX-2 expression at baseline correlates with pCR. This trial indicates that panitumumab may enhance the response of IBC patients to neoadjuvant chemotherapy.

The definitive role of EGFR-targeted therapy will be further determined by an ongoing randomized phase 2 study of carboplatin and paclitaxel with and without panitumumab in TN-IBC patients (ClinicalTrials.gov Identifier: NCT02876107; A Randomized Phase II Study of Neoadjuvant Carboplatin/Paclitaxel (CT) Versus Panitumumab/Carboplatin/Paclitaxel (PaCT) Followed by Anthracycline-Containing Regimen for Newly Diagnosed Primary Triple-Negative Inflammatory Breast Cancer.) [41]. This trial plans to recruit 72 patients and randomize them into two arms. In Arm A, patients receive panitumumab as a single agent in the window period followed by weekly panitumumab and paclitaxel and triweekly carboplatin for a total of four cycles. Patients in Arm B receive the same regimen as those in Arm A but without panitumumab. In both arms, this treatment will be followed by treatment with doxorubicin and cyclophosphamide for 4 cycles and then surgery. The pCR, disease-free survival, and overall survival (OS) rates, as well as the safety and tolerability of treatment regimens, will be determined.

Neratinib is a pan-EGFR receptor tyrosine kinase inhibitor that interacts with the catalytic domain of EGFR, HER2, and HER4. A phase II study of neratinib, pertuzumab, and trastuzumab with paclitaxel followed by doxorubicin and cyclophosphamide in HER2+ primary IBC, and neratinib with paclitaxel followed by doxorubicin and cyclophosphamide in HR+/HER2− primary IBC is ongoing (ClinicalTrials.gov Identifier: NCT03101748; Table 1) [42]. This study will determine the efficacy of neratinib with paclitaxel and with or without pertuzumab and trastuzumab in IBC and metastatic breast cancer.

## 3. HER2

Unlike other ErbB family members, HER2 has no known EGF-like ligands, and its activation for intracellular signaling pathways relies on the heterodimerization with other receptors in the ErbB family. The overexpression of HER2 through *HER2* (*ERBB2*) gene amplification or transcriptional deregulation plays important roles in many cancer types, including non-IBC and IBC. About 40% of IBC patients are HER2+, which is higher than the percentages of patients with LABC and non-T4 breast cancer patients [13]. However, HER2+ status is not a prognostic factor for IBC patients [13]. Fujii et al. [43] and Kogawa et al. [44] showed that the ratio of HER2 to centromere enumerator probe 17 on fluorescence in situ hybridization (FISH), also called the HER2 FISH ratio, is an independent predictor of high pCR in HER2+ LABC treated with neoadjuvant trastuzumab-containing regimens. However, the HER2/FISH ratio was not associated with pCR, recurrence-free survival, or OS in HER2+ IBC patients treated with neoadjuvant chemotherapy with or without trastuzumab [45].

The US Food and Drug Administration-approved HER2-targeted therapies for HER2+ breast cancer include trastuzumab and pertuzumab (anti-HER2 monoclonal antibodies); lapatinib, neratinib, and tucatinib (small molecule tyrosine kinase inhibitors); and T-DM1 (an antibody–drug conjugate). Chemotherapy combined with trastuzumab is the standard of care for HER2+ IBC patients. Trastuzumab prevents HER2 dimerization with HER receptors and inhibits phosphoinositide 3-kinase (PI3K) signaling and cell proliferation by inducing G1-phase cell cycle arrest [43]. Trastuzumab also engages in antibody-dependent cell-mediated cytotoxicity. A retrospective study showed that trastuzumab with preoperative treatment regimens produced high pCR rates in HER2+ IBC patients, but there was a risk of recurrence in the chest wall and relatively early recurrence in the brain [44]. The NOAH trial (ISRCTN86043495, http://www.isrctn.com/) was an open-label phase 3 trial in which newly diagnosed HER2+ LABC or IBC patients were treated with neoadjuvant chemotherapy consisting of doxorubicin, paclitaxel, cyclophosphamide, methotrexate, and fluorouracil with or without trastuzumab (Table 1). The study showed that trastuzumab significantly improved event-free survival and was well tolerated [46].

Pertuzumab inhibits the pairing of the HER2/HER3 heterodimer and abrogates HER2 intracellular signaling. It also mediates antibody-dependent cell-mediated cytotoxicity. NeoSphere (ClinicalTrials.gov Identifier: NCT00545688), an open-label phase 2 study to evaluate the efficacy of pertuzumab and trastuzumab in HER2+ IBC patients (Table 1), showed significantly higher pCR in patients treated with pertuzumab, trastuzumab, and docetaxel compared with those treated without pertuzumab [47].

Lapatinib inhibits the tyrosine kinase activity of both EGFR and HER2 [48]. A single-agent lapatinib in HER2+ IBC patients who had disease progression resulted in a 39% overall response rate and a 20.9-week median duration of response (ClinicalTrials.gov Identifier: NCT00105950; A Phase II Study to Evaluate the Efficacy, Safety and Pharmacodynamics of Lapatinib in Patients With Relapsed or Refractory Inflammatory Breast Cancer; Table 1) [49]. In a neoadjuvant setting, lapatinib followed by lapatinib plus paclitaxel in IBC patients showed a 78.6% combined clinical response rate based on Response Evaluation Criteria in Solid Tumors [50] and clinically evaluable skin disease criteria (Table 1) [51].

Although HER2-targeted therapy with trastuzumab and lapatinib is effective for patients with HER2+ IBC, resistance to both treatments develops within a year after the initiation of treatment. Lee et al. [52] showed that the combination of a histone deacetylase type I inhibitor, entinostat, with lapatinib synergistically inhibited proliferation in HER2+ breast cancer cells, including IBC SUM190 cells. Entinostat also sensitized trastuzumab- or lapatinib-resistant HER2-overexpressing cells to single-agent treatment with trastuzumab or lapatinib [53]. These results suggest the potential of combining entinostat with HER2-targeted therapies to improve the treatment of patients with HER2+ breast cancer. A phase 1b study was conducted in patients with HER2+ breast cancer to evaluate the combination of entinostat and lapatinib with or without trastuzumab (ClinicalTrials.gov Identifier: NCT01434303; Phase I and Phase I Trastuzumab Cohort Study of Entinostat, Lapatinib and Trastuzumab in Patients With HER2-Positive Metastatic Breast Cancer in Whom Trastuzumab Has Failed; Table 1). This trial defined the maximum tolerated dose for entinostat and showed that the combination treatment was safe [54]. A phase II study is needed to assess the clinical efficacy of entinostat in combination with HER2-targeted therapy in IBC.

HER2 also has non-amplification alterations, including single base substitution and insertion mutations, in breast cancer [52]. HER2 somatic mutations have been identified in breast cancer patients, and the majority of these mutations can drive tumorigenesis and are related to either resistance or sensitivity to HER2 inhibitors [55]. HER2 genomic alterations have been identified in 26% of IBC patients [56]. Using multiplex genome sequencing, Matsuda et al. [57] identified a higher frequency of *HER2* somatic mutations such as V777L, L755S, and D769Y in IBC, especially in the HR+ subtype, compared with non-IBC patients. V777L and D769Y, but not L755S, have been reported as activating mutations in breast cancer patients, and L755S mutation produced lapatinib resistance; however, the effects of these mutations have not been studied in IBC patients [55]. A clinical trial of neratinib with or without fulvestrant is ongoing in patients with metastatic breast cancer with *HER2* mutation but not amplification (ClinicalTrials.gov Identifier: NCT01670877; A Phase II Study of Neratinib Alone and in Combination With Fulvestrant in Metastatic HER2 Non-amplified But HER2 Mutant Breast Cancer.) [58]. One case report showed that a patient with *HER2* non-amplification IBC with L755S mutation in the kinase domain and S310F mutation in the extracellular domain responded to a lapatinib-based HER2-targeted therapy [59]. These findings suggest a potential opportunity to target *HER2* mutations and warrant clinical trials in IBC patients.

## 4. Inflammatory Pathways JAK/STAT, NF-ĸB, and COX-2

Inflammation plays crucial roles in promoting tumorigenesis and aggressiveness in IBC. The overexpression of inflammatory cytokines/chemokines, including TNFα, IL-6, and IL-8, have been reported in IBC cells and patient samples [57,60]. The JAK/STAT, NF-κB, and COX-2 inflammatory pathways are important in the progression of IBC. Here, we will summarize the current research findings of these pathways in IBC and the related inflammatory cytokines/chemokines.

### 4.1. JAK/STAT

#### 4.1.1. Biological Functions of JAK/STAT Pathway in IBC

JAK is a family of nonreceptor tyrosine kinases that includes JAK1, JAK2, JAK3, and TYK2. JAK family proteins associate with the intracellular domain of cytokine receptor and transduce cytokine-mediated signals together with the STAT pathway [61]. The JAK/STAT pathway contributes to many cellular processes, including immunity, cell growth, cell death, and differentiation. STAT activation is associated with the formation of various cancers, including melanoma, prostate cancer, non-IBC, and IBC. The hyperactivation of JAK2 signaling and STAT signaling along with the strong infiltration of macrophages was reported in IBC specimens with mammalian target of rapamycin (mTOR) activation [62]. Both the PI3K/mTOR and JAK/STAT pathways are strongly activated in IBC patient tumor tissues, suggesting a potential combination strategy of targeting both pathways in IBC [57]. Researchers led by Polyak et al. [17,18] showed that samples from IBC patients were enriched in CD44+/CD24− CSCs and activated phosphor-STAT3 (pSTAT3) cells, and 40% of CD44+/CD24− cells were positive for pSTAT3, suggesting STAT pathway activation in IBC CSCs. They also demonstrated that the inhibition of JAK2 reduced the proliferation and tumor growth of pSTAT3+ IBC cells in vitro and in vivo.

JAK/STAT signaling also regulates the IBC TME. For example, JAK/STAT signaling mediates the effect of M2 macrophages on the radiosensitivity of IBC cells [63]. IL-6, a major mediator of inflammation, induces the expression of STAT3 target genes and drives tumor growth and/or survival. IL-6/JAK2/STAT3 signaling can down-modulate the antitumor immunity of tumor-infiltrating immune cells because STAT3 negatively regulates the functions of neutrophils, natural killer (NK) cells, effector T cells, and dendritic cells. IL-6 is significantly upregulated in IBC patients compared with non-IBC patients [57,64]. The activation of JAK2/STAT3 signaling by IL-6 affects the IBC TME. A study by Wolfe et al. [65] showed that M2 macrophage-educated mesenchymal stem/stromal cells had elevated IL-6 secretion and increased IBC cells’ ability to invade and form mammospheres. The researchers further showed that inhibiting the recruitment of TAMs decreased pSTAT3 expression and IL-6 secretion within the TME, leading to delayed IBC tumor formation and reduced skin invasion and local recurrence. These results demonstrated that IL-6 mediates the tumor-promoting influences of the IBC TME.

#### 4.1.2. Targeting JAK/STAT in IBC

The activation of JAK2/STAT3 signaling in IBC and its role in mediating the crosstalk between IBC cells and the TME suggest that targeting this pathway may benefit patients with IBC. Two clinical trials of ruxolitinib, a dual JAK1/2 inhibitor, are underway in IBC patients. A Phase 1/2 study of ruxolitinib combined with preoperative chemotherapy is conducted in TN-IBC patients. This study will find the maximum tolerated dose of ruxolitinib when administrated with paclitaxel (Phase 1) and evaluate the efficacy of ruxolitinib combined with paclitaxel in TN-IBC patients (Phase 2) (ClinicalTrials.gov Identifier: NCT02041429; Phase II Study of Combination Ruxolitinib (INCB018242) With Preoperative Chemotherapy for Triple Negative Inflammatory Breast Cancer Following Completion of a Phase I Combination Study in Recurrent/Metastatic Breast Cancer; Table 1) [66]. Another randomized Phase 2 study of ruxolitinib combined with preoperative chemotherapy is conducted in newly diagnosed TN-IBC patients (ClinicalTrials.gov Identifier: NCT02876302; Phase II Study Of Combination Ruxolitinib (INCB018424) With Preoperative Chemotherapy For Triple Negative Inflammatory Breast Cancer; Table 1) [67]. In this clinical trial, 64 patients will be recruited, and the efficacy of ruxolitinib in combination with paclitaxel followed by standard chemotherapy with doxorubicin and cyclophosphamide, as well as the effect on the IL-6/JAK/STAT pathway, will be evaluated. Both clinical trials are pending results.

### 4.2. NF-ĸB

The activation of NF-κB plays a major role in promoting the unusual phenotype and aggressiveness of IBC. The NF-κB pathway is transcriptionally more active in IBC than in non-IBC [21]. IBC patient samples overexpress a large number of NF-κB target genes [20]. Lerebours et al. [19] measured the mRNA levels of 60 NF-κB-related genes in 35 patients with IBC, 22 with stage IIB and III non-IBC, and 24 with non-IBC and distant metastasis. The researchers found that 58% of NF-κB-related genes were upregulated in IBC compared with non-IBC, and these upregulated genes play roles in apoptosis, immune response, proliferation, tumor promotion, and angiogenesis. This study further identified a five-gene signature that matched patient outcomes, including NF-κB-related genes *IL-8* and vascular endothelial growth factor (*VEGF*). Van Laere et al. [68] further showed that NF-κB activation is linked to the loss of estrogen receptor expression in IBC, which is due to EGFR and/or ErbB overexpression.

NF-κB signaling contributes to the self-renewal of IBC. Kendellen et al. [69] showed that IBC SUM149 cells have activated NF-κB signaling, which is required for SUM149 cells to self-renew in vitro and to form xenograft tumors efficiently in vivo through the stimulation of EMT and the upregulation of IL-1β and IL-6.

The NF-κB pathway induces the expression of a variety of pro-inflammatory cytokines and chemokines and regulates inflammasomes. IL-6 and IL-8 are two cytokines regulated by the NF-κB pathway and are hyperactivated in IBC. Debeb et al. [70] showed that IBC SUM149 and SUM190 cells secreted significant amounts of both IL-6 and IL-8 compared with non-IBC cell lines. Inhibition of the NF-κB pathway by using tetrathiomolybdate, a specific copper chelator, or by transfecting a dominant-negative IκBα vector reduced the secretion of VEGF, fibroblast growth factor 2 (FGF2), IL-1α, IL-6, and IL-8 in vitro and SUM149 tumor growth and angiogenesis in vivo [65,71]. These results suggest that suppressing NF-κB signaling could inhibit IBC tumor growth, motility, and angiogenesis.

### 4.3. COX-2

COX-2 is overexpressed in a wide range of malignancies, including colon, bladder, prostate, pancreatic, and breast cancers, and it is associated with tumor growth and invasiveness [72]. IBC patients have increased expression of the prostaglandin I2 synthase gene *PTGIS*, which encodes an enzyme located downstream of COX-2 [20]. IBC cell lines also have higher levels of PGE2 and PGF2α, two enzymatic products of COX-2, than do non-IBC cell lines [22]. High COX-2 expression correlates with worse OS and higher nuclear grade in IBC patients [22]. In addition, the COX-2 pathway contributes to invasiveness and the CSC population in IBC [22]. As a key inflammatory molecule in IBC, COX-2 is functionally linked to other signaling pathways. The expression of COX-2 is regulated by the EGFR pathway and is correlated with EGFR expression in IBC specimens. COX-2 also mediates CSC regulation by the EGFR pathway through nodal signaling in IBC [22].

Several drugs inhibit COX enzymes. Celecoxib, a nonsteroidal anti-inflammatory drug, targets COX-2 and inhibited invasion and EMT in SUM149 cells. It also reduced SUM149 tumor growth and the expression of COX-2, PGE2, and PGF2α in vivo [22]. An alternative approach to target COX-2 is to block the binding of PGE2 to its prostanoid receptors, which consist of EP1, EP2, EP3, and EP4. PEG2 and the EP4 agonist, PGE2 alcohol, stimulated the proliferation and invasion of SUM149 cells, whereas an EP4 antagonist or EP4 knockdown inhibited these processes [73].

It is worth noting that the COX-2 pathway has emerging roles in modulating the tumor immune microenvironment. COX-2-derived PGE2 has been shown to mediate the crosstalk between colonic tumor cells and macrophages [74], induce the accumulation and activation of myeloid-derived suppressor cells [68,69], and promote the tumor growth of mutant BrafV600E mouse melanoma cells by suppressing immunity and fueling tumor-promoting inflammation. The depletion of COX-2 or prostaglandin E synthases switches the tumor inflammatory profile to anticancer immune pathways. Moreover, the inhibition of COX-2 synergizes with PD-1 blockade [70]. These data indicate the critical role of COX-2 and PGE2 in modulating the TME to an immunosuppressive status. Given that the TME drives the aggressive phenotype of IBC, it is important to investigate the crosstalk between the COX-2 pathway and TME and the efficacy of targeting both COX-2 and immune checkpoints in IBC.

## 5. Axl

### 5.1. Biological Functions of Axl Pathway in IBC

Axl belongs to the Tyro3-Axl-Mer family of receptor tyrosine kinases. Axl signaling regulates several biological processes, including cell proliferation, migration, angiogenesis, and differentiation. An aberrant overexpression of Axl has been reported in a variety of malignancies, such as breast, colorectal, lung, and ovarian cancers and melanoma. The overexpression or activation of Axl signaling promotes tumorigenesis, EMT, and tumor angiogenesis and is associated with poor prognosis and a high risk of metastasis [75]. It also contributes to resistance to conventional therapies in chronic myeloid leukemia [76], acute myeloid leukemia [77], lung cancer [78,79], esophageal adenocarcinoma [80], and colon cancer [81], as well as HER2-targeted agents lapatinib and trastuzumab in breast cancer [82]. Axl participates in the crosstalk with other receptor tyrosine kinases such as VEGFR, EGFR, MET, and platelet-derived growth factor receptor (PDGFR) through heterodimerization, which is one of the underlying mechanisms of Axl-related drug resistance. A recent study showed that Axl expression correlates with resistance to anti-PD-1 immunotherapy, suggesting a contribution of Axl to resistance to immunotherapies [83].

Axl expression correlates with the decreased survival of patients with triple-negative breast cancer (TNBC) [84,85]. Axl is also highly expressed in all IBC cell lines and is identified as a functional partner of oncogenic protein tazarotene-induced gene 1 (TIG1) in IBC [14]. TIG1 and Axl expression are positively correlated in primary IBC patient tissues, and the binding of TIG1 to Axl inhibits the proteasome-dependent degradation of Axl and supports the Axl signaling pathway in IBC cells [14]. Furthermore, Axl depletion reduced the proliferation and motility of IBC cells [14]. The inactivation of Axl signaling by the Axl inhibitor SGI-7079 and a more potent inhibitor TP-0903 (Ueno NT, unpublished results) significantly inhibited the proliferation, soft agar anchorage-independent growth, and invasion of IBC cells [14].

Axl is also expressed in various tumor-infiltrating immune cells, such as T cells, NK cells, B cells, and macrophages. It suppresses antigen presentation and produces myeloid-supporting cytokines and chemokines, resulting T-cell exclusion [86]. The genetic deletion of Axl enhanced T-cell infiltration up to 20-fold and enhanced the efficacy of radiotherapy and checkpoint immunotherapy [86]. Several preclinical studies indicated the enhanced efficacy of combining anti-checkpoint molecules with Axl inhibitors, including BGB324, TP-0903, and ONO-9330547, in breast and colon cancers [87,88,89]. Axl is essential for the development of human NK cells [90]. The inhibition of Axl and family members Tyro3 and Mer efficiently enhanced NK cell activity and thus reduced murine mammary cancer and melanoma metastases [91]. This family of proteins also plays a role in macrophage polarization and efferocytosis [92,93]. A study showed that *Axl* transcript levels are higher in M2-like than M1-like human monocyte-derived macrophages [94]. TNBC cells with *Axl* expression can polarize human macrophages toward an M2-like phenotype and modulate the expression of pro-tumor cytokines and chemokines [95]. The cytokine-dependent activation of Tyro3-Axl-Mer signaling inhibits Toll-like receptor (TLR) and TLR-induced cytokine-receptor cascades, which leads to an intrinsic feedback inhibition of both TLR- and cytokine-driven immune responses [96]. Cheng et al. [97] showed that the inactivation of Axl using TP-0903 reduced the polarization of M2 macrophages. Thus, targeting Axl could have a significant impact on antitumor immunity.

### 5.2. Targeting Axl in IBC

As described above, Axl inhibitors SGI-7079 and TP-0903 reduced IBC cell proliferation and invasion. Our group also showed that TP-0903 significantly reduced the tumor growth of IBC SUM149 and BCX-010 xenografts (unpublished data). In these xenograft mouse models, TP-0903 not only inhibited Axl signaling in tumor cells but also affected immunosuppression in the TME (unpublished data), suggesting that TP-0903 has dual effects on IBC tumors and the TME. Kumagai et al. [98] also showed that TP-0903 treatment decreased myeloid-derived suppressor cells in the spleen, increased the infiltration of and activated dendritic cells in the tumor, and enhanced the effect of an immune checkpoint inhibitor in an immune checkpoint inhibitor-resistant TNBC mouse model, 4T1. TP-0903 also reduced the tumor growth of KRAS-mutant colorectal cancer models, HCT-116, and a patient-derived xenograft model [99].

There are currently two clinical trials of TP-0903, a Phase 1 study in patients with advanced solid tumors (ClinicalTrials.gov Identifier: NCT02729298; A Phase 1a / 1b, First-in-human, Open-label, Dose-escalation, Safety, Pharmacokinetic, and Pharmacodynamic Study of Oral TP-0903 Administered Daily for 21 Days to Patients With Advanced Solid Tumors.) [100] and a Phase 1/2 study in patients with previously treated chronic lymphocytic leukemia (CLL) (ClinicalTrials.gov Identifier: NCT03572634; A Combined Phase 1/2 Study to Investigate the Safety, Pharmacokinetics, Pharmacodynamics, and Clinical Activity of TP-0903 in Patients With Previously Treated Chronic Lymphocytic Leukemia (CLL).) [101]. These clinical studies will identify the maximum tolerated dose, safety profile, and recommended phase II dose of TP-0903 in patients with solid tumors and previously treated CLL, respectively. The dual effects of TP-0903 on IBC tumor cells and the TME warrant a clinical study to determine its therapeutic efficacy in IBC patients.

## 6. JNK

The JNK signaling pathway regulates many biological processes, such as cell growth, differentiation, survival, apoptosis, and inflammatory response. JNKs hyperactivate in multiple cancer cell lines and tissues, including TNBC [102], hepatocellular carcinoma [103], and glioblastoma [104]. JNK1 and JNK2 can act as either tumor promoters or tumor suppressors in different cancer types. JNK is highly activated in basal-like TNBC [105]. Suppressed JNK activity is associated with longer OS in patients with infiltrating ductal breast cancer [106]. Xie et al. [107] also reported that TNBC samples had a higher expression of both total and phosphorylated c-Jun, a substrate of the JNK pathway, compared with samples from non-TNBC patients. They also showed that high *c-Jun* gene expression correlates with a shorter disease-free survival of primary TNBC patients [102].

*c-Jun* expression is also significantly upregulated in IBC compared with non-IBC patients [64]. Xie et al. [107] reported that JNK knockdown by small interfering RNA (siRNA) or inactivation by JNK inhibitor JNK-IN-8 suppressed proliferation, migration, invasion, tumor growth, and CSC phenotypes through NOTCH signaling in TNBC and IBC cells. These results support the notion that JNK signaling regulates IBC aggressiveness via promoting CSC phenotypes. However, a controversial study showed that the inhibition of N-myristoyltransferase 1 (NMT1) triggered reactive oxygen species and endoplasmic reticulum stress, which led to activation of the JNK pathway and autophagy and resulted in a reduction of proliferation of TNBC cells, including IBC SUM149 cells [108]. The researchers also showed that JNK inhibitor SP600125 abrogated the suppressive effect of NMT1 knockdown on SUM149 cell proliferation, implying a tumor-suppressive role of JNK signaling in IBC under oxidative stress and endoplasmic reticulum stress. Further studies are needed to elucidate the context-dependent role of JNK signaling in IBC, which will provide an opportunity to develop JNK-targeted therapy in IBC.

## 7. VEGF

Angiogenesis is a vital physiologic process that contributes to the growth, proliferation, and metastasis of most solid tumors [97,98]. In IBC, the intratumoral vascular area is enlarged [109], and most IBC patients at diagnosis have axillary lymph node involvement from the invasion of pre-existing lymph vessels and tumor-induced lymph angiogenesis [1]. IBC tumor samples have higher mRNA expression of angiogenic factors, including *VEGF*, *Flt-1*, *Ang1*, *Ang2*, and *Tie2*, than do non-IBC tumor samples [23,24]. A study by Colpaert et al. [110] indicated that both angiogenesis and high expression of E-cadherin may contribute to the metastatic phenotype of IBC. IBC patient tissues have high stromal VEGF-A expression, which is an independent predictor of poor breast cancer-specific survival and disease-free survival [111].

Several studies have evaluated agents that target VEGF in patients with IBC. Bevacizumab is a humanized monoclonal antibody to VEGF. In a pilot clinical study of 21 patients with LABC or IBC, bevacizumab inhibited VEGFR activation and vascular permeability and induced tumor cell apoptosis (Table 1) [112]. BEVERLY-2 was a phase 2 study in patients with primary HER2+ IBC (ClinicalTrials.gov Identifier: NCT00717405; An Open Label Study to Assess the Rate of Pathological Complete Response in Patients With Primary Inflammatory HER2-positive Breast Cancer Treated With Avastin + Herceptin Based Chemotherapy; Table 1) [113]. In this trial, 52 patients received four 3-week cycles of neoadjuvant fluorouracil, epirubicin, cyclophosphamide, and bevacizumab followed by four 3-week cycles of docetaxel, trastuzumab, and bevacizumab. Following resection, patients received adjuvant radiation therapy, trastuzumab, and bevacizumab. The pCR rate was 63.5% after neoadjuvant therapy, suggesting that preoperative therapy with bevacizumab, trastuzumab, and chemotherapy was effective. This trial also showed that high MMP2 and low MMP9 serum levels at baseline were associated with better survival outcomes in patients with HER2+ IBC who received neoadjuvant chemotherapy that included bevacizumab and trastuzumab [114]. However, another trial, BEVERLY-1 (ClinicalTrials.gov Identifier: NCT00820547; Phase II Study Evaluating the Efficacy and Tolerance of Bevacizumab (Avastin) in HER2- Inflammatory Breast Cancer; Table 1), indicated that the addition of bevacizumab to neoadjuvant and adjuvant chemotherapy conferred no clinical benefit to non-metastatic HER2− IBC patients [107]. Longer follow-up and the identification of predictive biomarkers for bevacizumab treatment are needed.

Tie2 kinase, a receptor of angiopoietins, has an important function in tumor growth, survival, and metastasis. A Phase 1b/2 study of rebastinib (DCC-2036), a selective and potent inhibitor of Tie2, in combination with paclitaxel in patients with advanced or metastatic solid tumors, including IBC, is ongoing (ClinicalTrials.gov Identifier: NCT03601897; An Open-Label, Multicenter, Phase 1b/2 Study of Rebastinib (DCC-2036) in Combination With Paclitaxel to Assess Safety, Tolerability, and Pharmacokinetics in Patients With Advanced or Metastatic Solid Tumors; Table 1) [115].

Pazopanib, an angiogenesis inhibitor, targets both VEGF and PDGF receptors. The combination of pazopanib and lapatinib in HER2+ LABC patients showed an increase in the objective response rate compared with lapatinib alone in the VEG20007 study [116,117]. Based on this result, a randomized phase 2 trial of lapatinib combined with pazopanib versus lapatinib alone was conducted in patients with relapsed HER2+ IBC (ClinicalTrials.gov Identifier: NCT00558103; A Randomized, Multicenter, Phase III Study Comparing the Combination of Pazopanib and Lapatinib Versus Lapatinib Monotherapy in Patients With ErbB2 Over-expressing Inflammatory Breast Cancer; Table 1). However, the combination did not improve rates of overall response and progression-free survival. It also had increased toxicity [118].

## 8. RhoC GTPase

RhoC GTPase is a member of the Rho family of proteins, which includes RhoA, RhoB, and RhoC. RhoC GTPase, an important regulator of cytoskeletal reorganization during cellular motility, is overexpressed in IBC tumor samples [16]. It produces a tumorigenic effect and recapitulates the highly invasive phenotype of IBC through the MAPK and PI3K/Akt pathway [119,120,121]. RhoC GTPase plays a role in modulating angiogenesis by inducing the production of VEGF and bFGF as well as inflammatory molecules IL-6 and IL-8 [121]. RhoC GTPase can be regulated by WISP3 in IBC. The dysregulation of WISP3 can upregulate RhoC GTPase and enhance the aggressiveness of IBC [11].

The Rho GTPase family can regulate glutaminase activity [122]. The RhoC GTPase family is a potent regulator of both glutamine and N-acetylaspartate metabolism in IBC SUM149 cells, revealing a novel role in regulating tumor cell metabolism [110]. RhoC GTPase also contributes to the TAM-promoted metastatic phenotype of IBC. Macrophage-conditioned media was shown to prime IBC cells to be highly migratory, but this effect could be abrogated by RhoC GTPase knockout, indicating a role of RhoC GTPase in regulating the TME’s effect on IBC cells and further supporting RhoC as a potential therapeutic target for interventions to inhibit IBC metastasis. Indeed, a novel RhoC antagonist delayed the onset of the primary tumor in a PyMT-RhoC transgenic mouse model and reduced primary tumor growth and lung metastasis compared with control mice [123]. Farnesyl transferase inhibitors (FTIs), previously shown to modulate tumor growth in Ras-transformed tumor cells, were able to reverse the phenotype induced by RhoC in IBC cells [120]. However, in a Phase 1/2 trial of tipifarnib, an orally available FTI, in combination with preoperative doxorubicin and cyclophosphamide in 22 HER2− IBC patients, showed that only one patient achieved pCR [112]. The clinical relevance of targeting RhoC GTPase in IBC needs further investigation.

## 9. Conclusions

IBC is the most lethal type of breast cancer. Although many efforts have been made, the unique biology of IBC compared with non-IBC has not been fully revealed. As summarized in this review, the biological functions of several activated or overexpressed signaling pathways in the progression and aggressiveness of IBC have been investigated. The efficacy of targeting these pathways in inhibiting tumor cell growth and metastasis shown in pre-clinical studies indicates that these pathways are potential therapeutic targets for patients with IBC. Other biological events, such as the attenuation of the TGFβ pathway and the genomic alteration of proteins controlling the cell cycle [Myc, cyclin dependent kinases 4 and 4 (CDK4 and CDK6)], have been reported in IBC [56,124,125]. Further investigations are needed to determine their biological functions and therapeutic roles in IBC. For clinical translation, treatments targeting the EGFR and JAK2 pathways are probably the most advanced to date. However, the true biological roles of these pathways remain unclear pending more definitive clinical data. Recent evidence indicates that the TME is the driver of IBC’s clinical phenotype and aggressiveness. Further understanding of the crosstalk between these signaling pathways and the TME, and of the underlying mechanisms, is needed. Such understanding may lead to the identification of novel molecules from the TME that could enhance the efficacy of targeted therapy drugs and further translate these findings into clinical trials for patients with IBC.

## Figures and Tables

**Table 1:** Clinical trials in inflammatory breast cancer.

Drug Studied	Target(s)	Combined Agents	Type of Study	Patient Population	Trial Identification No.
Panitumumab	EGFR	Carboplatin, nab-paclitaxel, 5-fluorouracil, epirubicin, and cyclophosphamide	Phase 2, single-arm	Primary HER2− newly diagnosed IBC	NCT01036087
Panitumumab	EGFR	Carboplatin, nab-paclitaxel, 5-fluorouracil, epirubicin, and cyclophosphamide	Phase 2, randomized	TN-IBC	NCT02876107
Neratinib	EGFR, HER2, and HER4	Neratinib, pertuzumab, and trastuzumab with paclitaxel	Phase 2	HER2+ IBC; HR+/HER2− IBC	NCT03101748
Trastuzumab	HER2	Trastuzumab, doxorubicin, paclitaxel, cyclophosphamide, methotrexate, 5-fluorouracil, and tamoxifen	Phase 3	Newly diagnosed HER2+ locally advanced breast cancer or IBC	ISRCTN86043495
Pertuzumab and trastuzumab	HER2	Pertuzumab, trastuzumab, and docetaxel	Phase 2	Locally advanced, inflammatory or early stage HER2+ breast cancer	NCT00545688
Lapatinib	HER2	Lapatinib	Phase 2	HER2+ IBC	NCT00105950
Lapatinib	HER2	Lapatinib and paclitaxel	Phase 2	Newly diagnosed IBC	EGF102580
Lapatinib	HER2	Lapatinib, entinostat, with or without trastuzumab	Phase 1b	HER2+ breast cancer	NCT01434303
Ruxolitinib	JAK1/2	Ruxolitinib with preoperative chemotherapy	Phase 1/2	TN-IBC	NCT02041429
Ruxolitinib	JAK1/2	Ruxolitinib, paclitaxel, doxorubicin, and cyclophosphamide	Phase 2, randomized	TN-IBC	NCT02876302
Bevacizumab	VEGF	Bevacizumab, doxorubicin, and docetaxel	Interventional	Inflammatory and locally advanced breast cancer	NCT00717405
Bevacizumab	VEGF	Bevacizumab, fluorouracil, epirubicin, cyclophosphamide, and docetaxel	Phase 2	Non-metastatic HER2− IBC	NCT00820547
Rebastinib	Tie2	Rebastinib and paclitaxel	Phase 1b/2	Advanced or metastatic solid tumors including IBC	NCT03601897

Abbreviations: IBC, inflammatory breast cancer; nab, nanoparticle albumin-bound; TN-IBC, triple-negative inflammatory breast cancer.
